# Emerging Infectious Diseases: Threats to Human Health and Global Stability

**DOI:** 10.1371/journal.ppat.1003467

**Published:** 2013-07-04

**Authors:** David M. Morens, Anthony S. Fauci

**Affiliations:** National Institute of Allergy and Infectious Diseases, National Institutes of Health, Bethesda, Maryland, United States of America; Duke University Medical Center, United States of America

The inevitable, but unpredictable, appearance of new infectious diseases has been recognized for millennia, well before the discovery of causative infectious agents. Today, however, despite extraordinary advances in development of countermeasures (diagnostics, therapeutics, and vaccines), the ease of world travel and increased global interdependence have added layers of complexity to containing these infectious diseases that affect not only the health but the economic stability of societies. HIV/AIDS, severe acute respiratory syndrome (SARS), and the most recent 2009 pandemic H1N1 influenza are only a few of many examples of emerging infectious diseases in the modern world [1]; each of these diseases has caused global societal and economic impact related to unexpected illnesses and deaths, as well as interference with travel, business, and many normal life activities. Other emerging infections are less catastrophic than these examples; however, they nonetheless may take a significant human toll as well as cause public fear, economic loss, and other adverse outcomes.

## Determinants of Emergence and Reemergence

Historical information as well as microbial sequencing and phylogenetic constructions make it clear that infectious diseases have been emerging and reemerging over millennia, and that such emergences are driven by numerous factors (Table 1). Notably, 60 to 80 percent of new human infections likely originated in animals, disproportionately rodents and bats, as shown by the examples of hantavirus pulmonary syndrome, Lassa fever, and Nipah virus encephalitis [2]–[4]. Most other emerging/reemerging diseases result from human-adapted infectious agents that genetically acquire heightened transmission and/or pathogenic characteristics. Examples of such diseases include multidrug-resistant and extensively drug-resistant (MDR and XDR) tuberculosis, toxin-producing *Staphylococcus aureus* causing toxic shock syndrome, and pandemic influenza [1]–[10].

**Table 1:** Some major factors that underlie disease emergence and reemergence [2], [5].

The Microbial Agent	The Human Host	The Human Environment
Genetic adaptation and change	Human susceptibility to infection	Climate and weather
Polymicrobial diseases	Human demographics and behavior	Changing ecosystems
	International trade and travel	Economic development and land use
	Intent to harm (bioterrorism)	Technology and industry
	Occupational exposures	Poverty and social inequality
	Inappropriate use of antibiotics	Lack of public health services
		Animal populations
		War and famine
		Lack of political will

Although precise figures are lacking, emerging infectious diseases comprise a substantial fraction of all consequential human infections. They have caused the deadliest pandemics in recorded human history, including the Black Death pandemic (bubonic/pneumonic plague; 25–40 million deaths) in the fourteenth century, the 1918 influenza pandemic (50 million deaths), and the HIV/AIDS pandemic (35 million deaths so far) [6], [9].

## Definition and Concepts

Two major categories of emerging infections—newly emerging and reemerging infectious diseases—can be defined, respectively, as diseases that are recognized in the human host for the first time; and diseases that historically have infected humans, but continue to appear in new locations or in drug-resistant forms, or that reappear after apparent control or elimination [1]. Emerging/reemerging infections may exhibit successive stages of emergence. These stages include adaptation to a new host [11], an epidemic/pathogenic stage, an endemic stage, and a fully adapted stage in which the organism may become nonpathogenic and potentially even beneficial to the new host (e.g., the human gut microbiome) or stably integrated into the host genome (e.g., as endogenous retroviruses). Although these successive stages characterize the evolution of certain microbial agents more than others, they nevertheless can provide a useful framework for understanding many of the dynamic relationships between microorganisms, human hosts, and the environment.

It is also worth noting that the dynamic and complicated nature of many emerging infections often leaves distinctions between emerging and reemerging infections open to question, leading various experts to classify them differently. For example, we describe as “reemerging” new or more severe diseases associated with acquisition of new genes by an existing microbe, e.g., antibiotic resistance genes, even when mutations cause entirely new diseases with unique clinical epidemiologic features, e.g., Brazilian purpuric fever [12]. Similarly, we refer to SARS as an emerging disease a decade after it disappeared, and apply the same term to the related MERS (Middle East Respiratory Syndrome) β coronavirus which appeared in Saudi Arabia in late 2012 [13].

## Examples of Newly Emerging Infectious Diseases

The most salient modern example of an emerging infectious disease is HIV/AIDS, which likely emerged a century ago after multiple independent events in which the virus jumped from one primate host to another (chimpanzees to humans) and subsequently, as a result of a complex array of social and demographic factors, spread readily within the human population. AIDS was not recognized as a distinct entity until 1981 [6], [9], after its initial detection among certain risk groups, such as men who have sex with men, recipients of blood products, and injection drug users. It was soon apparent, however, that the disease was not restricted to these groups, and indeed, the bulk of HIV infections globally has resulted from heterosexual transmission that has been heavily weighted within the developing world, particularly sub-Saharan Africa where a number of factors were responsible for this rapid spread; chief among these were human movement along truck routes accompanied by a high level of commercial sex work, inadequate public health infrastructures, poverty, and social inequality.

Other examples of disease emergences [1]–[10] include SARS, which emerged from bats and spread into humans first by person-to-person transmission in confined spaces, then within hospitals, and finally by human movement between international air hubs. Nipah virus also emerged from bats and caused an epizootic in herds of intensively bred pigs, which in turn served as the animal reservoir from which the virus was passed on to humans. The 2009 H1N1 pandemic influenza virus emerged from pigs as well, but only after complex exchanges of human, swine, and avian influenza genes [14]. H5N1 influenza emerged from wild birds to cause epizootics that amplified virus transmission in domestic poultry, precipitating dead-end viral transmission to poultry-exposed humans. Additional examples are many [1]–[10]; however, the variables associated with emergences are unique for each and typically complex.

## Examples of Reemerging Infectious Diseases

Most of the important reemerging infectious disease agents first appeared long ago, but have survived and persisted by adapting to changing human populations and to environments that have been altered by humans. Dengue virus and West Nile virus (WNV), distantly related flaviviruses, serve as good examples. They have been spread by geographic movement of humans in association with the mosquito vectors for the diseases. For example, dengue came to the Americas in association with the slave trade of earlier centuries. In this regard, slaves infected by mosquitoes in Africa presumably brought the infection to the Americas by seeding the mosquito population upon arrival [15]. Similarly, WNV came to the United States in 1999 when an infected human, bird, or mosquito came by air travel from the Middle East to the Western Hemisphere, providing a source for introduction of infection to New World mosquitoes and birds. Pathogenic strains of dengue have also spread back from Southeast Asia to the Western Hemisphere, as has a major mosquito vector, *Aedes albopictus*. Unlike most arboviruses, which are partly or completely host-restricted, WNV has become adapted to multiple mosquito and avian species, a major factor in increasing its opportunity to infect humans. The lack of additional hosts undoubtedly drove the mosquitoes that are the vectors of dengue and the dengue virus itself to favor adapting to humans and to their behaviors and environments. The association of dengue with *Aedes* mosquitoes that live in and around human habitations mean that crowding, poor sanitation, and poverty provide ideal environments for transmission to humans [15]. Host immunity factors are also thought to be involved in the severe/fatal form of dengue known as dengue shock syndrome [15].

Other non-arboviral examples of emerging infections abound. For example, cholera has repeatedly reemerged over more than two centuries in association with global travel, changing seasons, war, natural disasters, and conditions that lead to inadequate sanitation, poverty, and social disruption. Emergences of disease caused by community- and hospital-acquired *Clostridium difficile* and methicillin-resistant *Staphylococcus aureus* (MRSA) have been driven by increased and/or inappropriate use of antibiotics, and some hospital-acquired organisms such as MRSA have now moved into community transmission. The global emergence of plasmid-spread NDM-1 (New Delhi β-lactamase) Gram-negative pan-resistant organisms, linked to global antibiotic use and inadequate antibiotic stewardship, medical tourism, economic globalization, and other aspects of modern life, has prompted calls for development of international control mechanisms [16] that are applicable to a number of emerging bacterial diseases in the developing and developed world. Drug resistance mutations have also caused the reemergences of certain pathogens such as multidrug-resistant and extensively drug-resistant tuberculosis, drug-resistant malaria, and numerous bacterial diseases such as vancomycin-resistant enterococci. Fungi have made significant contributions to disease emergence as well. In Africa, cryptococcal disease has already surpassed tuberculosis as a leading cause of death [17]. Other examples of fungal emergence include comorbidities in HIV-infected individuals (17), *Cryptococcus gattii* epidemics in predominantly healthy persons in the U.S. [18], [19], and a 2012 U.S. nationwide epidemic of *Exserohilum rostratum* infections associated with contaminated pharmaceutical products [20].

## Will We Ever Eliminate Emerging Infectious Diseases?

While it has become possible to eradicate certain infectious diseases (smallpox and the veterinary disease rinderpest), and to significantly control many others (dracunculiasis, measles, and polio, among others), it seems unlikely that we will eliminate most emerging infectious diseases in the foreseeable future. Pathogenic microorganisms can undergo rapid genetic changes, leading to new phenotypic properties that take advantage of changing host and environmental opportunities. Influenza viruses serve as a good example of emerging and reemerging infectious agents in their ability to rapidly evolve in response to changing host and environmental circumstances via multiple genetic mechanisms. New “founder” influenza viruses [21] appear periodically, cause a pandemic, raise widespread population immunity, and then, in response to human immune pressures, evolve and persist for decades using multiple genetic evolutionary mechanisms to sustain continual immune escape. The 1918 influenza pandemic virus is one example: over the past 95 years, its descendants have evolved continually by antigenic drift, intra-subtypic reassortment, and antigenic shift, the latter producing new pandemics in 1957 and 1968 [14]. Even the genetically complex 2009 pandemic H1N1 influenza virus is a descendant of the 1918 virus [14]. Such continuous genetic hyper-evolution forces us to develop new influenza vaccines containing new antigens on an annual basis.

In the meantime, new human diseases keep emerging. As noted, in late 2012 the novel MERS coronavirus emerged in Saudi Arabia [13], and in early 2013 a new H7N9 avian influenza virus became epizootic in Eastern China, causing 132 spillover infections of humans (as of June 7, 2013), with 28 percent case fatality [10], [22]. Its pandemic potential, if any, remains to be determined. Whether or not such outbreaks become more widespread, they nonetheless attract global attention and require significant international effort to monitor and contain. Microbial advantages can be met and overcome only by aggressive vigilance, ongoing dedicated research, and rapid development and deployment of such countermeasures as surveillance tools, diagnostics, drugs, and vaccines.

We appear to be entering a new era in which several important emerging, reemerging, and stable infectious diseases are becoming better controlled (e.g., hepatitis B, rabies, *Haemophilus influenzae* type B, and even to some extent HIV/AIDS). However, our success in stopping the many new emerging diseases that will inevitably appear is not assured. We have many tools in our armamentarium, including preparedness plans and stockpiles of drugs and vaccines. But each new disease brings unique challenges, forcing us to continually adapt to ever-shifting threats [1]–[10], [23]. The battle against emerging infectious diseases is a continual process; winning does not mean stamping out every last disease, but rather getting out ahead of the next one.
